# Accumulated Plastic Deformation Monitoring of Cement Sheath Interface Using Fiber-Optic Bragg Gratings

**DOI:** 10.3390/s26113572

**Published:** 2026-06-04

**Authors:** Yongqin Cheng, Yanxin Jin, Xiran Xia, Hui Xie, Shuoqiong Liu, Jiyun Shen

**Affiliations:** 1 State Key Laboratory of Chemical Safety, Qingdao 266000, China; 2 SINOPEC Research Institute of Safety Engineering Co., Ltd., Qingdao 266000, China; 3 CNPC Engineering Technology R&D Co., Ltd., Beijing 102206, China

**Keywords:** well integrity, cement sheaths cumulative plastic strain, mechanical properties, non-destructive testing, Fiber Bragg Grating

## Abstract

**Highlights:**

**What are the main findings?**

- Quantified the signal drift characteristics (1.5–2% error) of FBG sensors under cyclic loading; revealed its core source is bonding/anchoring loosening.
- Revealed the evolution difference in cement sheath plastic strain between uncon-strained triaxial tests and constrained full-scale CCFS.

**What are the implications of the main findings?**

- Proposed an optimized parallel winding + end fixing layout method to improve long-term monitoring reliability.
- Clarified the inhibition mechanism of stress redistribution on plastic accumulation, and defined the conservatism of traditional triaxial tests for cement sheath sealing capacity evaluation.

**Abstract:**

Accurately characterizing the accumulated plastic deformation of the cement sheath is essential for evaluating wellbore integrity. Fiber Bragg Grating (FBG) technology, noted for its strong immunity to external interference, is employed for in situ monitoring under harsh downhole conditions. This study investigates the accumulated plastic deformation behavior of set cement through uniaxial cyclic loading–unloading tests and proposes a real-time, high-precision, and non-destructive monitoring scheme by integrating FBG sensors into the Casing–Cement–Formation system (CCFS). The results reveal that under uniaxial conditions, cumulative plastic strain increases with stress amplitude, with the plastic strain in a single cycle capable of reaching up to 0.2%. Under identical conditions, FBG measurements exhibit a drift phenomenon, resulting in an error margin of approximately 1.5–2%. Furthermore, within the CCFS, plastic strain exhibits linear accumulation during the initial 2–5 cycles, followed by a deceleration in the accumulation rate. This deceleration is attributed to the redistribution of internal stress induced by plastic strain accumulation. Notably, the addition of silica fume and latex significantly mitigates this deformation. Collectively, these findings validate the effectiveness of FBG technology for downhole integrity assessment and offer a pathway for early failure detection and targeted maintenance.

## 1. Introduction

The progression towards deep and unconventional resources exposes the cement sheath to harsh downhole environments. Significant internal pressure fluctuations, driven by drilling fluid density adjustments during multi-stage drilling and high-pressure hydraulic fracturing, subject the cement sheath to severe cyclic loading [1]. This dynamic stress environment poses a severe threat to long-term wellbore sealing integrity, frequently leading to Sustained Casing Pressure (SCP) [2].

**Figure 1:**
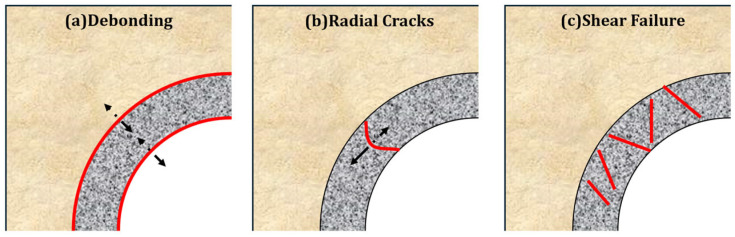
Three major types of cement failure.

As shown in Figure 1, cement sheath integrity loss manifests primarily in three forms: interfacial debonding (micro-annulus), bulk radial tensile cracking, and shear failure [3,4]. These failure modes are not independent but rather inherently interconnected. Under cyclic wellbore loading, failure typically initiates with localized interfacial plastic deformation, which then progressively develops into a connected micro-annulus, or, due to stress concentration, leads to bulk radial tensile or shear failure. This coupling effect introduces considerable complexity into the analysis of cement sheath integrity loss and underlies the ongoing controversy regarding the dominant mechanism by which cyclic loading leads to SCP. A large body of earlier triaxial testing studies generally attributed SCP to radial or tensile failure of the cement sheath [5,6]. However, in recent years, the application of high-resolution micro-CT and scanning electron microscopy (SEM) has demonstrated that, in many cases, no detectable macroscopic cracks exist within the cement matrix; instead, failure is caused by a micro-annulus with a thickness of only 1~10 μm, far below the resolution limit of conventional logging tools [7,8,9]. These discrepancies arise primarily from two causes: (1) scale mismatch, as most laboratory studies employ small-scale cement specimens (25 × 50 mm) [10] tested under fixed-amplitude stress cycles and therefore cannot replicate the complex boundary constraints and stress redistribution present in a full-scale Casing–Cement–Formation system (CCFS) [11]; and (2) monitoring technology limitations, since conventional sonic and ultrasonic logging can only detect macroscopic cracks (>100 μm), while strain gauges are highly susceptible to damage during cement hydration and introduce artificial weak interfaces [12,13]. Cumulative plastic strain represents the key intermediate variable linking cyclic loading to delayed cement sheath failure. However, owing to the constraints, the quantitative relationship between cumulative plastic strain and the different failure modes remains poorly understood.

FBG has emerged as a high-precision in situ monitoring technology with significant advancements across diverse fields, and have been successfully implemented in the oil and gas industry [14,15,16]. Compared with conventional logging methods, it offers distinct advantages in distributed and real-time monitoring capabilities [11]. FBG are periodic microstructured optical filters inscribed within the core of an optical fiber, which reflect a specific wavelength of incident light internally within the fiber core, referred to as the Bragg wavelength. In our previous work, we successfully demonstrated the preliminary real-time monitoring of strain and temperature variations both within the cement sheath and at its interfaces under laboratory conditions using this technology [17,18]. However, these experiments were primarily conducted under steady-state conditions and utilized single-point monitoring configurations, which cannot fully capture the influence of cement sheath geometric characteristics on interfacial accumulated plastic deformation under cyclic loading. Furthermore, multi-point sensor deployment using conventional techniques would inevitably alter the native stress distribution of the cement sheath.

Based on the above considerations, this study proposes a method for characterizing the accumulated plastic deformation of a full-scale cement sheath under cyclic loading, utilizing a CCFS and FBG technology, and demonstrates the advantages of FBG-based measurement of accumulated plastic deformation in the cement sheath. This work builds upon prior experimental investigations in which FBG sensors were employed to measure the interfacial bonding strength of the cement sheath. The key distinction is that the service condition of the FBG sensors in this study shifts from a steady-state regime to a cyclic compressive loading regime, thereby enabling the quantification of variations in FBG measurement accuracy under such dynamic conditions. Accordingly, a novel fiber design and layout scheme tailored to this service condition was developed. Furthermore, the advantages and limitations of different measurement technologies for downhole cement strain characterization were systematically compared. This study first outlines the selected cement slurry systems, curing protocols, and testing procedures. Subsequently, the accumulated plastic deformation behavior of various cement systems under different cyclic loading conditions is characterized, and comparisons are drawn between conventional methods and the new FBG-based approach. Critical factors influencing FBG performance are identified, and based on the acquired data, the limitations of conventional monitoring techniques are discussed. Finally, key directions for future research are proposed.

## 2. Experimental Setup

In this study, three distinct cement slurry systems were selected for testing, as detailed in Table 1: Neat API Class G cement(#1); a high-strength and high-elastic-modulus cement system(#2); and a low-strength and low-elastic-modulus cementitious system(#3). All specimens were cured at 70 °C for 72 h to ensure sufficient strength development.

**Figure 2:**
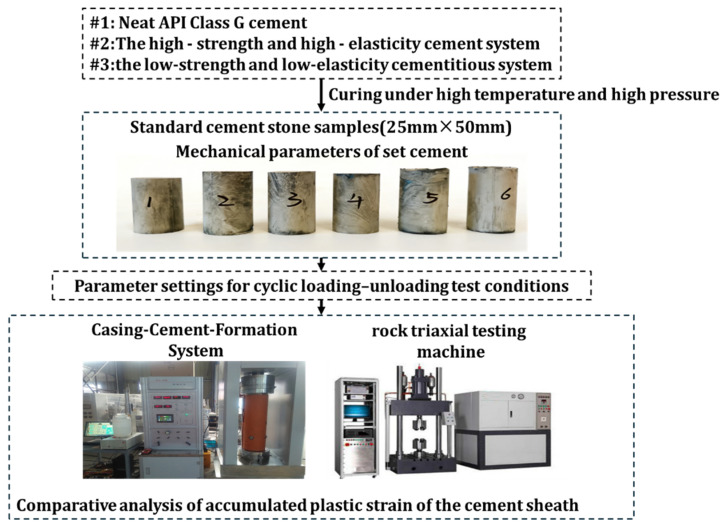
Test process and objectives.

Upon completion of curing, cylindrical specimens (25 × 50 mm) were prepared. We first conducted standard uniaxial and triaxial compression tests to obtain the mechanical properties of the cement systems, which were essential for defining the cyclic stress amplitudes. Subsequently, cyclic loading-unloading tests were carried out using a rock triaxial tester and a full-scale CCF simulator. To quantify the cumulative plastic strain under identical conditions, extensometers were employed for the small-scale specimens, while FBG sensors were utilized for the full-scale cement sheath. The overall testing procedure and research objectives are presented in Figure 2.

## 3. Mechanical Testing of Set Cement

### 3.1. Uniaxial and Triaxial Compression Tests

**Figure 3:**
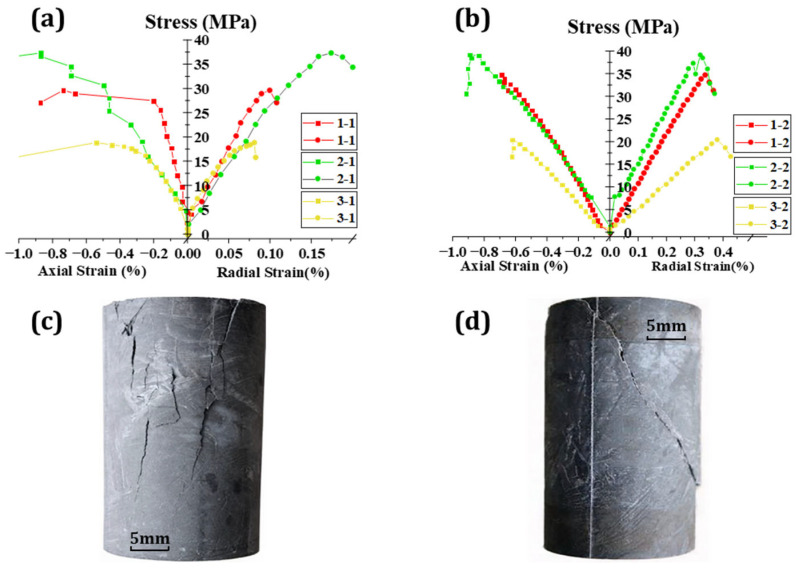
(**a**) Stress–strain curve under uniaxial compression. (**b**) Stress–strain curve under triaxial compression. (**c**) Failure modes of hardened cement stone specimen under uniaxial compression. (**d**) Failure modes of hardened cement stone specimen under triaxial compression.

The stress–strain curves for the different cement systems are presented in Figure 3a,b. Under uniaxial loading, a sharp decline in stress is observed immediately following the peak, exhibiting distinct elastic–brittle characteristics. Conversely, in triaxial compression tests, increasing the confining pressure significantly enhances both the peak strength and the resistance to deformation.

This enhancement is clearly reflected in the stress–strain data. For example, in System #1, the confining pressure elevated the peak strength from 28.92 MPa to 34.71 MPa, representing a 20% increase. According to Griffith’s strength theory, lateral confinement reduces the tensile stress concentration at the tips of micro-cracks, thereby raising the energy threshold for crack growth and inhibiting crack initiation [19,20]. This theoretical mechanism is consistent with the experimental observations. In uniaxial tests, where crack propagation is unconstrained by confining pressure, the specimens developed multiple interconnected fractures and a pulverized appearance (Figure 3c). In contrast, triaxial specimens failed along a single dominant shear fracture oriented at approximately 45°, with no other macroscopic cracks, indicating that the confining pressure successfully arrested the propagation of micro-cracks (Figure 3d). The detailed testing conditions and mechanical parameters are summarized in Table 2.

### 3.2. Quantification of Cumulative Plastic Strain in Set Cement Specimens

Cyclic loading tests were conducted using the rock triaxial testing machine. Each specimen was subjected to 10 loading-unloading cycles. The loading phase was performed under displacement control at a rate of 0.12 mm/min, while the unloading phase was conducted under force control at a rate of 0.5 kN/s. The upper stress limit for the cycles was determined based on the peak compressive strength (UCS) of the corresponding cement system. Specifically, stress levels were set at 0.4 UCS (Condition A), 0.6 UCS (Condition B), and 0.8 UCS (Condition C). These three loading conditions were designed to represent distinct service states of the cement sheath: (1) a stable elastic regime (Condition A), corresponding to normal wellbore pressure fluctuations; (2) a transitional damage regime (Condition B), approximating typical hydraulic fracturing loads at which plastic strain may begin to accumulate; and (3) an overload regime (Condition C), representing extreme pressure scenarios that drive the material into a state of rapid damage accumulation. The detailed parameters for each testing condition are summarized in Table 3.

**Figure 4:**
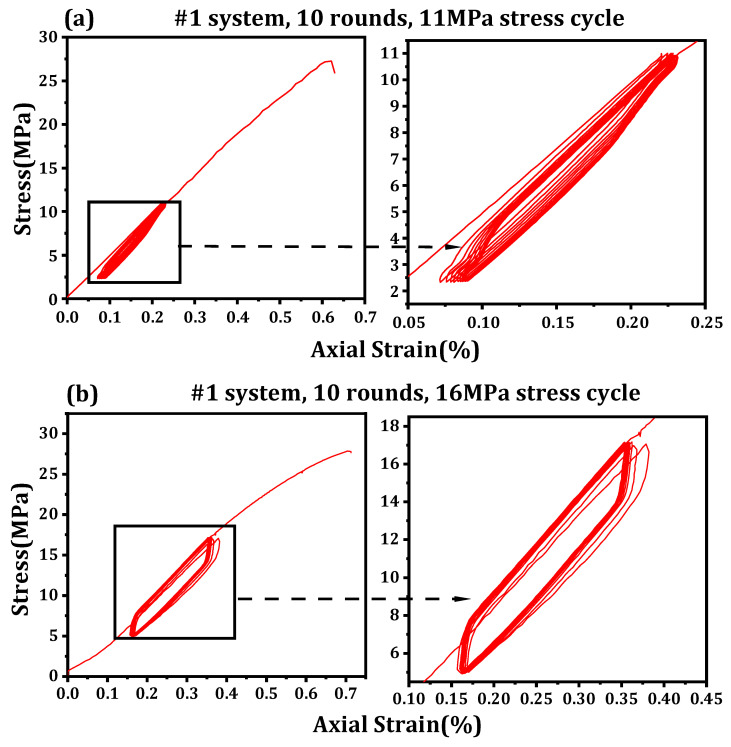

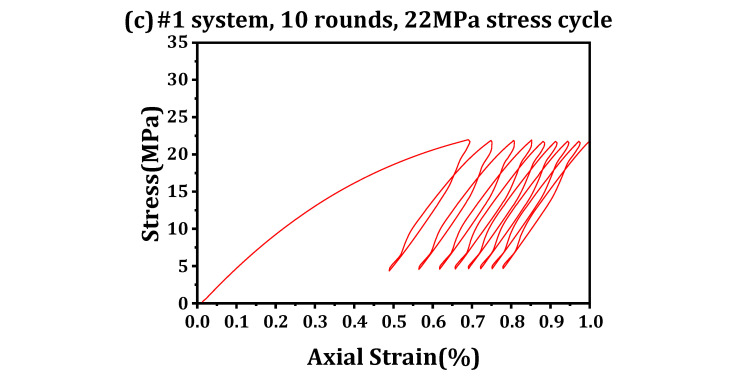
Plastic strain evolution in System 1 across different cyclic stress amplitudes: (**a**) An amount of 11 MPa stress cycle (Specimens 1–3), (**b**) 16 MPa stress cycle (Specimens 1–4), (**c**) 22 MPa stress cycle (Specimens 1–5).

**Figure 5:**
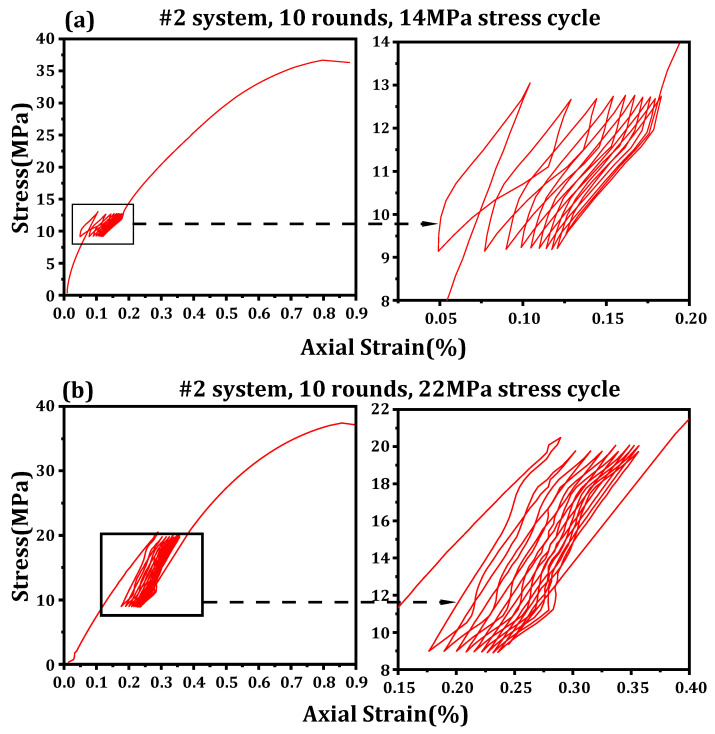

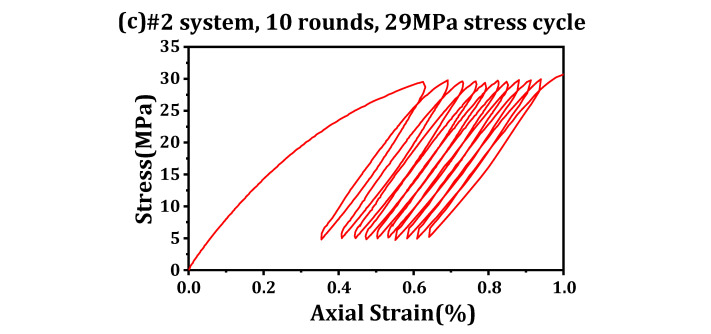
Plastic strain evolution in System 2 across different cyclic stress amplitudes: (**a**) A total of 14 MPa stress cycle (Specimens 2–3), (**b**) 22 MPa stress cycle (Specimens 2–4), (**c**) 29 MPa stress cycle (Specimens 2–5).

**Figure 6:**
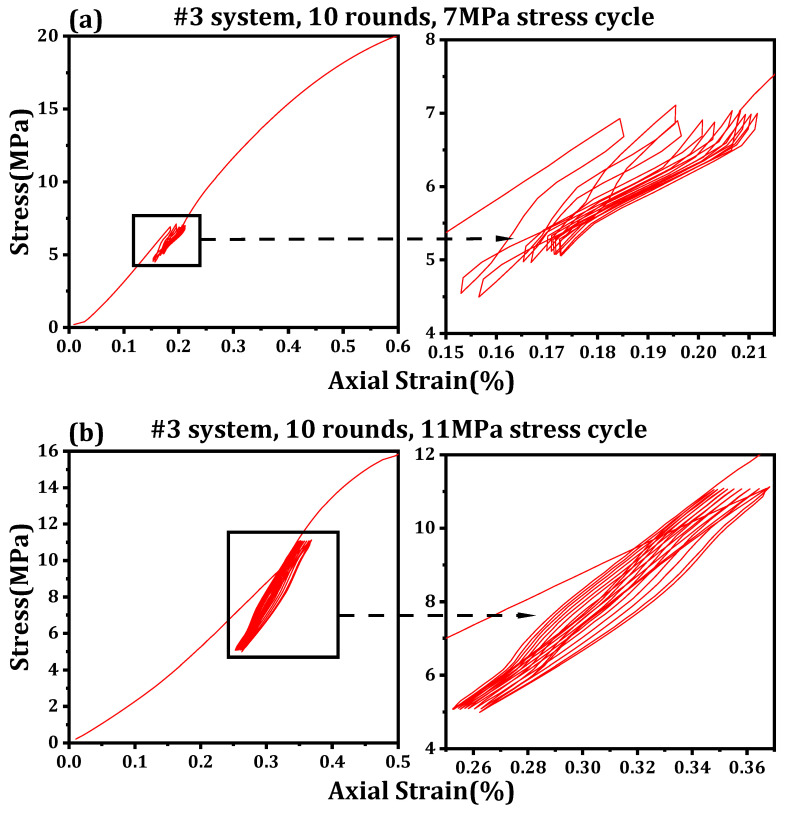

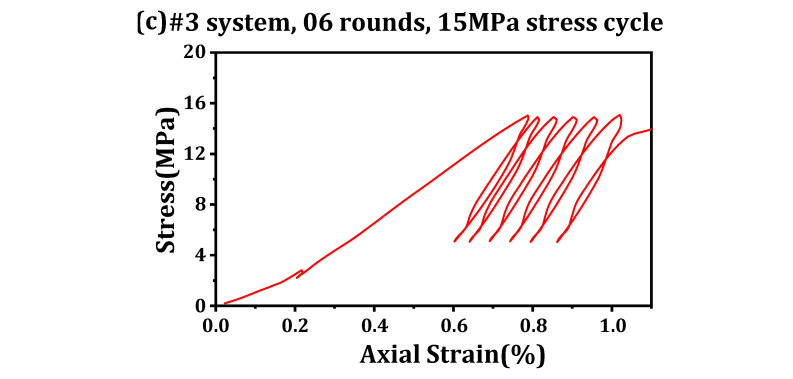
Plastic strain evolution in System 3 across different cyclic stress amplitudes: A total of (**a**) 7 MPa stress cycle (Specimens 3–3), (**b**) 11 MPa stress cycle (Specimens 3–4), (**c**) 15 MPa stress cycle (Specimens 3–5).

Figure 4, Figure 5 and Figure 6 illustrate the accumulation of plastic strain for the three cement slurry systems during stress cycling. It is evident that the stress–strain curves exhibit an approximately linear trend during the initial loading phase. Unloading commences immediately upon reaching the upper stress limit, followed by reloading once the stress decreases to the lower limit. Distinct hysteresis loops are formed between the reloading and unloading paths, with each cycle contributing to additional plastic strain. Furthermore, as the cumulative plastic strain increases, the hysteresis loops exhibit a progressive shift to the right.

**Figure 7:**
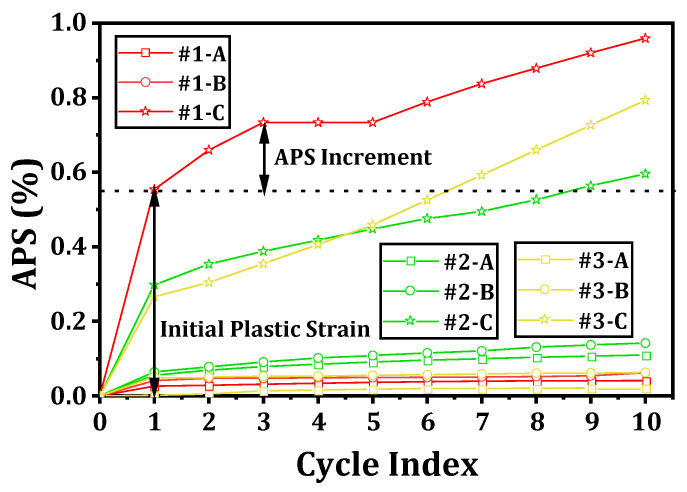
Accumulated plastic strain during 10 cycles.

The initial plastic strain is defined as the residual strain at the end of the first unloading phase, measured after the applied stress returns to the baseline level (lower stress limit). For each subsequent cycle, the plastic strain increment is defined as the difference in residual strain measured at the end of the unloading phase between the current cycle and the preceding cycle. Figure 7 illustrates the evolution of cumulative plastic strain with the number of loading cycles. It is evident that all three systems (#1–#3) exhibit a distinct ‘bilinear characteristic.’ Specifically, the first increment (initial plastic strain) accounts for the largest proportion of the total deformation. From the second cycle onwards, the subsequent increments show an approximately linear growth trend. Generally, under identical confining pressure, higher peak stress levels result in more pronounced plastic strain accumulation.

As illustrated in Figure 7, for System #1 under cyclic stress conditions of 0.4 and 0.6 times the peak strength (Conditions A and B), the applied stress remained below the yield strength. Consequently, the loading and unloading paths exhibited a near-linear behavior, resulting in negligible plastic strain ranging from 0.03% to 0.05%. In contrast, a distinct response was observed at 0.8 times the peak strength (Condition C). Here, the specimen entered the plastic deformation regime during the very first loading phase, accumulating a plastic strain of 0.3%. Plastic strain continued to accumulate over the subsequent eight cycles until brittle fracture occurred during the 10th cycle, at which point the peak strain reached 0.97%. These characteristics are consistent across Systems #2 and #3. For conditions 2-A, 2-B, 3-A, and 3-B, the maximum plastic strain remained below 0.1%. However, for specimens 2-C and 3-C, the plastic strain reached 0.2% in the initial cycle, with subsequent cycles showing pronounced accumulation, where the single-cycle strain increment exceeded 0.05%.

These results demonstrate that set cement behaves as a quasi-brittle porous material. During the initial loading phase, pre-existing internal micro-pores and micro-cracks undergo irreversible closure and compaction. Concurrently, if the stress level exceeds a certain threshold, initial micro-crack nucleation occurs within the cement matrix [17,18]. From the second cycle onwards, the material enters a stage of stable damage accumulation. In Condition B across all three systems, each loading–unloading cycle induces stress concentration at micro-crack tips, driving slow and steady crack propagation. Conversely, a shakedown limit is observed in Condition A. This arises because the low stress amplitude allows crack tips to become blunted by plastic zone development or arrested by residual compressive stresses, effectively halting further expansion [19,20,21]. The ‘linear growth’ observed in Conditions B and C is a classic manifestation of fatigue damage evolution, following a progression from damage accumulation to strain softening, and finally to sudden fracture. Condition C (#1-C, #2-C, #3-C) exhibited this process rapidly. During damage evolution, the equivalent elastic modulus degraded. Upon reaching a critical cumulative plastic strain, distributed micro-cracks transitioned from a dispersed state to coalescence, forming a macroscopic dominant fracture [22,23]. Once formed, the load-bearing capacity vanished instantaneously, resulting in catastrophic brittle collapse.

## 4. Full-Scale Quantification of Cumulative Plastic Strain

### 4.1. CCFS Assembly and Testing System

**Figure 8:**
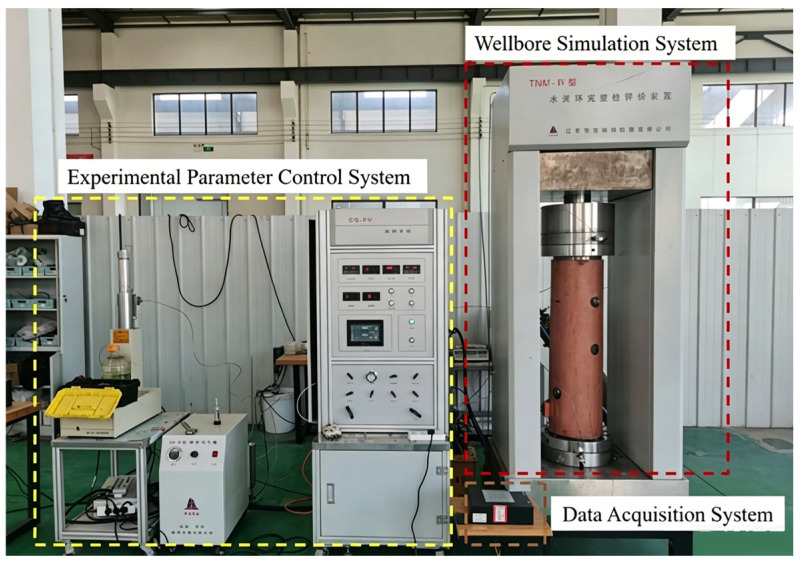
Schematic diagram of the full-size integrity evaluation device.

The downhole CCFS was simulated by creating a concentric steel pipe arrangement and injecting cement into the annular space. This experimental configuration has been previously utilized to monitor the bonding strength at the casing–cement interface under complex conditions involving temperature and pressure fluctuations [14]. The experimental apparatus consists of three main components: a wellbore simulation system, a data acquisition system, and an experimental parameter control system, as illustrated in Figure 8.

**Figure 9:**
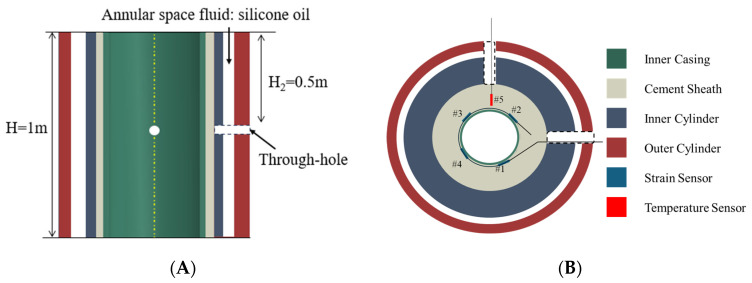
(**A**) Profile and (**B**) FBG sensors layout of the wellbore simulation system.

The wellbore simulation system, as shown in Figure 9A, is composed of an inner casing, a cement sheath, an inner cylinder, and an outer cylinder. The cement sheath is created by filling the annular space between the inner casing and the inner cylinder with cement and allowing it to solidify. The annular space between the inner cylinder and the outer cylinder is filled with silicone oil, which is heated by an internal heating rod to replicate a high-temperature downhole environment. The geometric dimensions and mechanical parameters of the system are detailed in Table 4.

**Figure 10:**
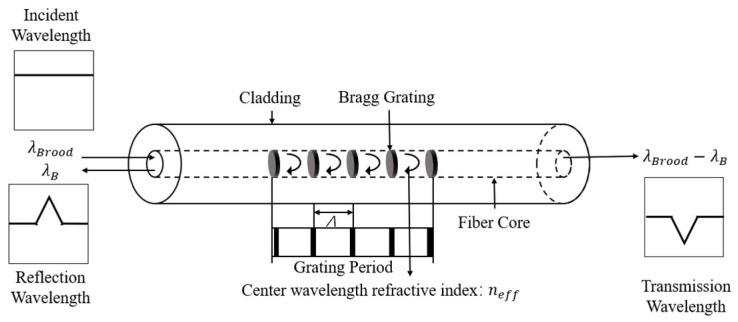
Schematic diagram of fiber optic sensor.

The data acquisition system encompasses an inner casing pressure gauge, an inner casing temperature gauge, an annular space temperature gauge, an annular space pressure gauge, as well as strain and temperature optical fibers. The inner casing pressure and temperature gauges are primarily used for the measurement of fluid pressure and temperature within the inner casing. The annular space temperature gauge is specifically designed to measure the temperature of the fluid in the outer annular space. Strain and temperature at the cement sheath interface are acquired through the strain optical fibers and temperature optical fibers, as depicted in Figure 9B. To enrich the dataset from a single experiment, one strain-sensing optical fiber with four measurement points and one temperature-sensing optical fiber were embedded in the cement sheath. FBG sensing technology enables quantitative measurements of strain and temperature by detecting the Bragg wavelength reflected or transmitted by gratings inscribed within optical fibers. Its fundamental operating principle relies on the fact that the Bragg wavelength is determined by two intrinsic parameters: the grating period and the effective refractive index of the fiber core (Figure 10). Any physical perturbation that modifies these two parameters will induce a measurable shift in the grating’s Bragg wavelength. Equation (1) quantifies the wavelength drift as a function of axial strain and temperature variations, and can be derived separately using established methods [21]. The FBG sensors used in this study were commercially manufactured (Shanghai Hankun Optoelectronic Technology Co., Ltd., Shanghai, China) and feature a single-ended fiber pigtail design. The sensor body has a diameter of 1 mm, and LC/APC optical connectors were used to interface the sensors with the interrogator. Each FBG sensor exhibits a grating reflectivity of ≥80%. The optical fiber interrogator employed was the HKA-M200 (Shanghai Hankun Optoelectronic Technology Co., Ltd., Shanghai, China).

(1) $$\frac{d\lambda_{B}}{\lambda_{B}}=K_{\varepsilon }\varepsilon_{zz}+K_{T}dT$$

where $d\lambda_{B}$ is wavelength drift, $\varepsilon_{zz}$ is an external axial strain, $\lambda_{B}$ is the central wavelength of a fiber optic grating, dT is the amount of temperature change, K; $K_{\varepsilon }$ is the sensitivity coefficient of fiber optic grating strain sensing, $K_{T}$ is the sensitivity coefficient of fiber optic grating temperature sensing.

According to the thick-wall theory, the correlation between the pressure on the steel pipe’s inner wall and the strain on the pipe’s outer wall can be expressed as:

(2) $$\Delta P_{i}=\frac{b^{2}-a^{2}}{2a^{2}}\left[\Delta \varepsilon_{\theta }E_{sp}+\Delta P_{c}\frac{b^{2}+a^{2}}{b^{2}-a^{2}}-2\mu \Delta P_{c}\right]$$

where $\Delta P_{i}$ is the change in internal casing pressure, MPa; *b* is the casing outer diameter, mm; *a* is the casing inner diameter, mm; $\Delta \varepsilon_{\theta }$ is the average change in tangential strain measured on the outer surface of the steel pipe, %; $E_{sp}$ is the Young’s modulus of the steel pipe, MPa; $\Delta P_{c}$ is the change in cement-casing interface stress, MPa; μ is the Poisson’s ratio of the steel pipe.

The experimental parameter control system is equipped with an inner casing pressure pump, an inner casing heating tube, and an annular space heating tube. The inner casing pressure pump and heating tube are used for the regulation of pressure and temperature within the inner casing. The annular space heating tube is employed for the temperature control within the annular space. The pressure control within the inner casing is achieved by the suction and expulsion of the fluid through the pressure pump, which facilitates both pressurization and depressurization within a range from 0 to 70 MPa with an accuracy of 0.1 MPa. The temperature control for both the inner casing and the annular space is executed by heating the fluid, with a control range from ambient temperature to 150.0 °C and an accuracy of 0.5 °C. In light of the temperature characteristics of this experiment, silicone oil has been selected as the filling fluid for both the inner casing and the annular space.

### 4.2. Testing Procedure

This study addresses two core tasks: validating the FBG measurement technique and quantifying the accumulated plastic deformation of the cement sheath. The former seeks to confirm the reliability and constraints of FBG sensors in the test setup, while the latter evaluates their suitability for downhole monitoring.

**Figure 11:**
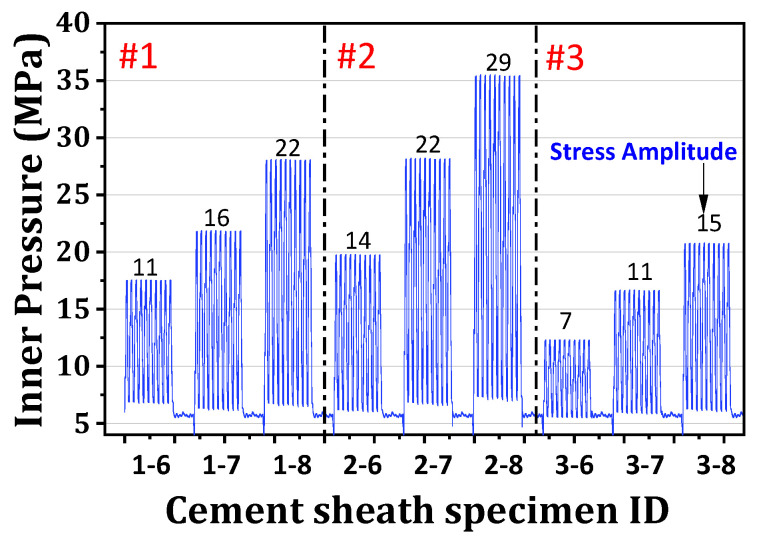
Internal pressure loading path.

After the validation phase, the water in the inner annulus was replaced by cement slurries (formulations per Table 1). The apparatus was sealed and subjected to curing at 70 °C for 72 h. Subsequently, cyclic loading was initiated with a baseline pressure of 5 MPa and stress amplitudes identical to the laboratory specimen tests. The protocol involved 10 cycles per pressure level, incorporating a 5 min holding period at peak and valley pressures. Figure 11 illustrates the specific loading scenarios for each system.

### 4.3. Validation Experiment

To evaluate the accuracy of the FBG measurement system, a series of validation experiments were conducted. In these tests, water was utilized as the annular fill medium instead of cement. The primary objective was to assess the feasibility of using FBG sensors for internal data acquisition within the CCFS under controlled and simplified conditions prior to conducting more complex experiments. The first validation test focused on parameter determination: the coefficients $E_{sp}$ and μ required for Equation (2) were derived by fitting the data obtained from the outer wall hoop strain of the inner casing, the inner casing pressure, and the annular pressure. The second validation test involved subjecting the inner casing to cyclic pressure variations. Based on the hoop strain measured by the FBG, the annular pressure was back-calculated using Equation (2) and compared with direct pressure gauge readings. The comparative results are presented in Figure 11.

**Figure 12:**
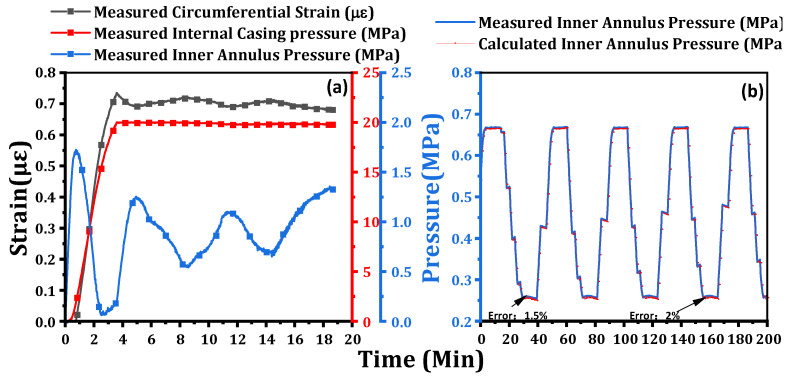
(**a**) Parameter acquisition experiment; (**b**) comparison between calculated values and pressure gauge measurements.

Based on the test results in Figure 12a, the coefficients E=204 GPa, μ=0.27 for Equation (1) were determined via data fitting. Utilizing these corrected parameters, the FBG response (depicted as the red curve in Figure 12b) showed high consistency with the inner annulus pressure readings throughout five alternating cycles. A two-sample variance test was conducted, yielding an F-statistic of 1.00003, which indicates no statistically significant difference in variance between the two datasets at the 0.05 significance level. However, a closer inspection reveals that in the low-pressure range, the error between the pressure gauge readings and the processed FBG data increased from 1.5% to 2%, exhibiting an increasing trend. This suggests a potential gradual drift in FBG response under cyclic conditions, a phenomenon that aligns with observations in Xiong Q [22]. The primary cause is attributed to the mechanical loosening of the adhesive agent responsible for ensuring tight contact between the optical fiber and the casing. Consequently, the bonding method and adhesive medium are identified as critical constraints for maintaining the long-term precision of FBG sensors under multi-cycle operating conditions.

**Figure 13:**
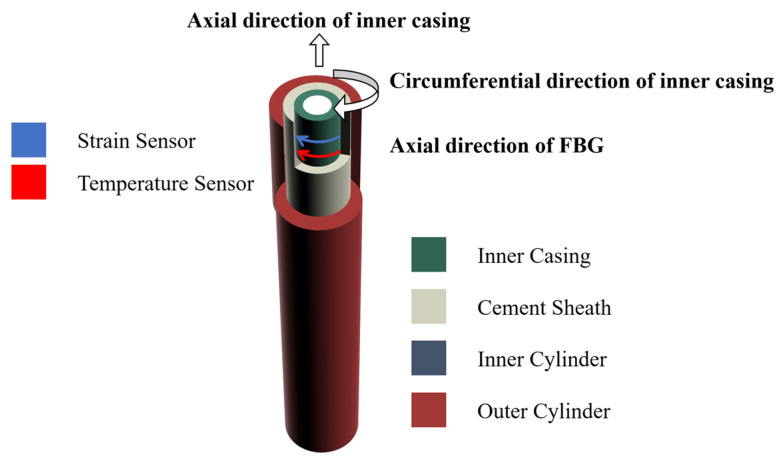
Schematic diagram of the spatial direction of the experimental system.

To ensure accurate measurements, the target measurement points on the outer wall of the inner casing must be meticulously cleaned with acetone when laying the strain-sensing optical fibers. Furthermore, as illustrated in Figure 12, the axial orientation of the strain-sensing FBG region must be precisely aligned along the circumferential direction of the outer surface of the inner casing. Accordingly, we adopted a parallel winding method with the fiber ends fixed, rather than a helical winding and fixing approach (Figure 13). This choice not only enhances coupling but also eliminates the influence of the winding angle on the measurements. After re-testing, the error growth phenomenon was significantly mitigated, with an error of ±10 με between measurement points across different cycles under the same pressure value.

### 4.4. Full-Scale Cyclic Loading Experiments

**Figure 14:**
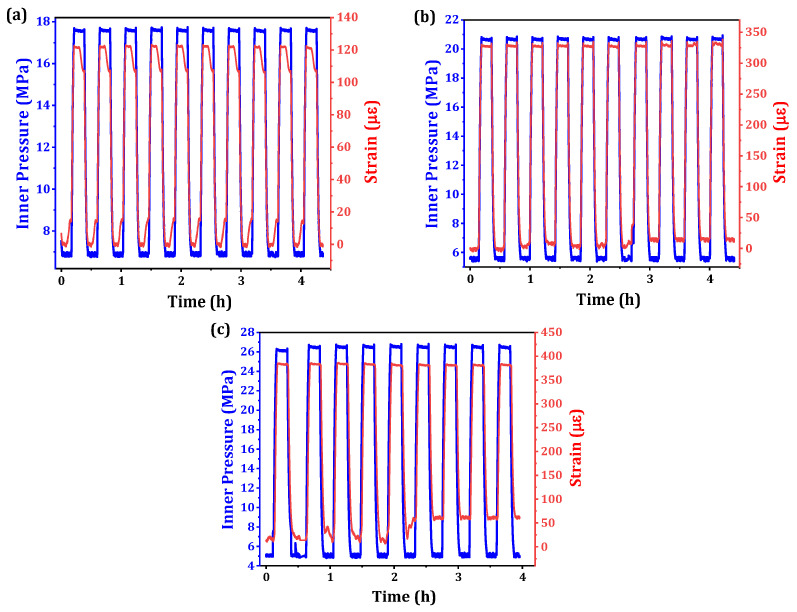
Circumferential strain variations in the cement sheath in System 1 under cyclic loading at 11~22 MPa: (**a**) Results at 11 MPa cyclic load (1-6), (**b**) results at 16 MPa cyclic load (1-7), and (**c**) results at 22 MPa cyclic load (1-8).

**Figure 15:**
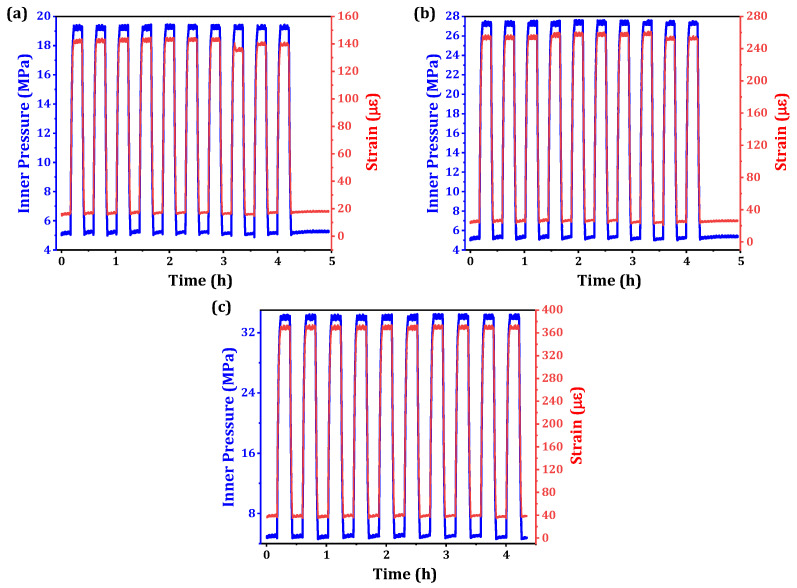
Circumferential strain variations in the cement sheath in System 2 under cyclic loading at 14~29 MPa: (**a**) Results at 14 MPa cyclic load (2-6), (**b**) results at 22 MPa cyclic load (2-7), and (**c**) results at 29 MPa cyclic load (2-8).

**Figure 16:**
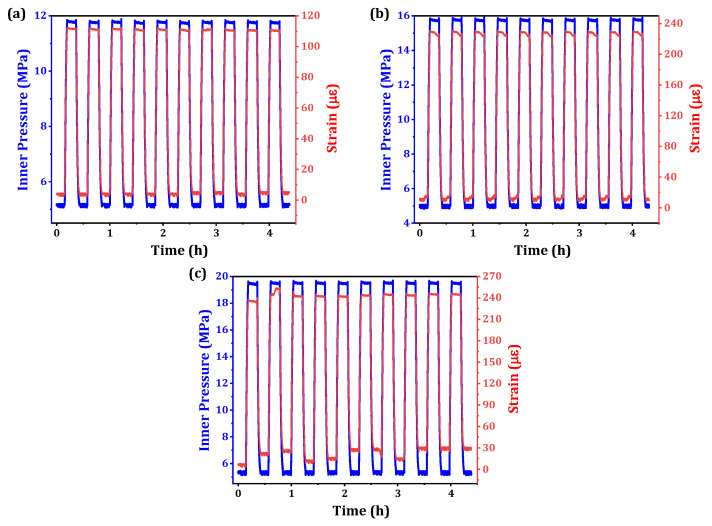
Circumferential strain variations in the cement sheath in System 3 under cyclic loading at 7~15 MPa: (**a**) Results at 7 MPa cyclic load (3-6), (**b**) results at 11 MPa cyclic load (3-7), and (**c**) results at 15 MPa cyclic load (3-8).

Figure 14, Figure 15 and Figure 16 illustrate the variations in hoop strain at different radial positions within the cement sheath under cyclic loading. These values represent the relative change, referenced to the strain recorded at the conclusion of cement setting. The results indicate that an increase in casing pressure induces tensile hoop strain at the cement sheath interface. Depending on the magnitude of the pressure increase, the hoop strain at the inner casing-cement interface ranges from 120 to 360 με. From a qualitative perspective, the strain curves at the bonding interface exhibit a trend consistent with the casing pressure variations, demonstrating the high sensitivity of the FBG sensing technique for strain measurement.

Figure 14a indicates that the neat cement system (#1) remained elastic at low pressures (1-6) but developed plastic strain at 16–22 MPa. The evolution curves (Figure 14b,c) show that for 1-7 and 1-8, strain grew linearly for the first 2–5 cycles before plateauing. Final strains at cycle 10 were 21 με and 63 με, respectively. Conversely, System #2 maintained excellent zonal isolation (Figure 15), and no plastic strain exceeding the measurement resolution was detected (i.e., plastic strain ≤ 10 με). System #3 displayed minor accumulation under high stress, capped at 31 με (Figure 16). These findings reveal a distinct discrepancy compared to the triaxial cyclic tests. While the full-scale assembly showed stabilization or minimal strain, the triaxial specimens were characterized by immediate onset and unabated linear accumulation of plastic strain from the second cycle onwards [17,23].

This difference likely stems from stress redistribution accompanying the accumulation of plastic strain. Relevant studies have explicitly demonstrated that plastic deformation of the cement sheath induces local stress redistribution, thereby altering the stress path and damage evolution rate under subsequent loading [24,25]. Strain mismatch at the casing-cement interface generates localized stress fields [26,27]. With each cycle, the accumulation of plastic strain alters the boundary interaction, causing the internal stress state to shift-a phenomenon distinct from the fixed-amplitude stress cycles applied in triaxial testing [4]. This dynamic evolution of the stress field mitigates the rate of strain accumulation compared to the unconstrained triaxial tests. However, sufficient accumulation of plastic deformation can still induce debonding, resulting in the creation of a micro-annulus and the subsequent failure of the system’s zonal isolation capacity.

In terms of plastic strain evolution, the full-scale cement sheath shares fundamental similarities with small-scale triaxial specimens, yet displays distinct behavioral differences. Under the highest stress level, System #1 accumulated 0.97% plastic strain in the triaxial test, compared with only 0.0063% in the CCFS; System #2 exhibited an initial plastic strain of 0.2% in the triaxial test, whereas no plastic strain exceeding the measurement resolution was detected in the CCFS; System #3 showed a single-cycle plastic strain increment exceeding 0.05% in the triaxial test, while the total cumulative plastic strain in the CCFS was merely 0.0031%. A key distinction is that the full-scale sheath undergoes indirect loading. Therefore, even when stress levels surpass the yield point, the system retains good sealing integrity initially, with plastic strain accumulating at the inner wall only after repeated cycling. Furthermore, systems incorporating additives sustained superior isolation performance even under high-cycle conditions. Specifically, the addition of silica fume and latex (System #2 and System #3) significantly inhibited plastic strain accumulation compared with the neat cement (System #1). This mitigating effect can be inferred to be associated with microstructural densification and pore refinement. Previous studies have demonstrated that silica fume and latex can reduce the porosity and enhance the strength and toughness of set cement through a micro-filling effect [28]. This suggests that assessments based exclusively on triaxial cyclic testing tend to be conservative, as they may not fully account for the beneficial structural constraints present in the actual wellbore, thereby yielding lower performance values than those realized in situ.

## 5. Conclusions

Characterizing accumulated plastic deformation is vital for wellbore integrity management. FBG offer a robust solution for in situ monitoring given their tolerance to extreme downhole environments. This study established and validated an FBG-based measurement methodology through both small-scale triaxial and full-scale assembly tests, successfully identifying key error sources. This work demonstrates the promise of FBG technology for downhole applications while noting necessary improvements. The main conclusions drawn from this study are as follows:

(1) Unlike triaxial tests, where plastic strain accumulates continuously from the onset, the CCFS exhibits plastic strain only at elevated casing pressures, maintaining elasticity at lower loads. This behavioral difference suggests that internal stress redistribution plays a critical role in full-scale assemblies. Consequently, further study on the evolution of cumulative plastic strain in constrained systems is required.
(2) This study demonstrates that proper sensor anchoring is critical for reliable FBG strain measurements under cyclic loading, as improper bonding can induce significant signal drift. The proposed parallel winding method effectively mitigates this drift and enhances measurement reliability.
(3) Increasing casing pressure generates radial compressive and hoop tensile strains. These strains are amplified when initial setting deformation is factored in, raising the failure risk. However, silica flour and latex additives effectively inhibited plastic accumulation, likely due to porosity reduction. Thus, initial strain and porosity must be included as key variables in cement sheath integrity analysis.
(4) This study focused solely on cyclic internal pressure fluctuations. In actual downhole conditions, the cement sheath is also subjected to non-uniform in situ stresses and thermal gradients. Future work will extend this sensing technique to investigate cement sheath integrity under non-uniform in situ stresses and high-temperature conditions, and to develop high-temperature–corrosion-resistant FBG packaging and protection solutions.

## Figures and Tables

**Table 1:** Composition of the cement slurry system.

Cement System Number	Cement Class	Additive	Water-Cement Ratio
#1	Class G (Shandong Huayin Special Cement Co., Ltd., Huayin, China)	-	0.44
#2	Class H (Shandong Huayin Special Cement Co., Ltd., Huayin, China)	silica fume (Qingdao Xiken Import and Export Co., Ltd., Qingdao, China) (35% by volume)	0.45
#3	Class G (Shandong Huayin Special Cement Co., Ltd., Huayin, China)	Latex (Qingdao Xiken Import and Export Co., Ltd., Qingdao, China) (30% by volume)	0.44

**Table 2:** Mechanical parameters of uniaxial and triaxial cement stone at room temperature.

Specimen Number	Confining Pressure	Yield Strength	Yield Strain	Peak Strength	Peak Strain	Young’s Modulus	Poisson’s Ratio
	MPa	MPa	%	MPa	%	GPa	
1-1	0	17.27	0.52	28.92	0.6	5.9	0.18
1-2	15	23.14	0.62	34.71	0.69	6.1	0.19
2-1	0	24.87	0.78	37.31	0.87	7.1	0.2
2-2	15	26.19	0.82	39.14	0.89	7.3	0.18
3-1	0	12.54	0.45	18.82	0.54	4.37	0.15
3-2	15	13.61	0.51	20.43	0.62	4.59	0.16

**Table 3:** Stress cycle test condition.

Cement System Number	Peak Strength	Peak Strength Under Cyclic Loading (Specimen Number)		
	MPa	MPa		
		A	B	C
#1	28.92	11(1-3)	16(1-4)	22(1-5)
#2	37.31	14(2-3)	22(2-4)	29(2-5)
#3	18.82	07(3-3)	11(3-4)	15(3-5)

**Table 4:** Dimensions and mechanical parameters of wellbore simulation system.

Parameters	Inner Casing		Cement Sheath		Inner Cylinder		Outer Cylinder	
	Outside Diameter	Thickness	Outside Diameter	Thickness	Outside Diameter	Thickness	Outside Diameter	Thickness
Width	139.7 mm	7.72 mm	165.1 mm	12.7 mm	195.1 mm	15 mm	245.1 mm	20 mm
Young’s modulus	210 GPa				210 GPa		210 GPa	
Poisson’s ratio	0.3				0.3		0.3	

## Data Availability

The data presented in this study are available on request from the corresponding author due to the data is considered company property.
